# Prevalence of Factors Related to Depressive Symptoms Among Married Individuals

**DOI:** 10.7759/cureus.49797

**Published:** 2023-12-01

**Authors:** Lokesh Krishnan, Gunjan Batra, Surbhi Batra, Alagirisamy Kuppusamy, Krishnan Gireesh, Faheem Vellekkat, Vivek Sanker

**Affiliations:** 1 Statistics, Periyar University, Salem, IND; 2 Information Systems and Security, Kennesaw State University, Kennesaw, USA; 3 Psychiatry, Atal Bihari Vajpayee Institute of Medical Sciences and Dr. Ram Manohar Lohia Hospital, New Delhi, IND; 4 Psychiatry, All India Institute of Medical Sciences, Jodhpur, IND; 5 Clinical Psychology, School of Behavioural Sciences, Trivandrum, IND; 6 Psychiatry, Indira Gandhi Medical College & Research Institute, Malappuram, IND; 7 General Surgery, Noorul Islam Institute of Medical Science (NIMS), Trivandrum, IND

**Keywords:** prevalence, married individuals, married, depressive symptoms, depression, machine learning

## Abstract

Background

There are multiple studies that indicate that the psychological well-being of a couple and their life satisfaction depend on the family and society. Various factors such as family, family values, marriage style, married life, and education have a great impact on people's lives both directly and indirectly. It is important to understand the effects of these factors on married individuals' lives that lead to depression so that appropriate measures can be taken for its prevention.

Objectives

This research aims to find the relationship of depressive symptoms among married individuals with various factors such as their marriage style, education, and having children.

Materials and methods

The study included 433 married individuals from Istanbul who met the criteria for depression. The early identification and prediction of depression in married individuals have been demonstrated to benefit significantly from machine learning techniques. In this study, we used decision tree (DT) and random forest (RF) predictive modeling techniques to create a model to predict the occurrence of depression among married individuals.

Results

The accuracy of the DT approach was found to be 80%, and the RF approach was 60%. Our results showed that as compared to conventional statistical methods, machine learning models performed better for classifying couples.

Conclusion

Future support systems that employ a range of data sources to identify individuals who are extremely susceptible to developing depression among married people may be developed using these effective models.

## Introduction

The middle-aged population is thought to be at their time of maturation, while the elderly are thought to be in their terminal phases of life. These stages denote the end of childrearing, the development and transmission of cultural norms and values to succeeding generations, as well as the understanding and consideration of the meaning of life in light of the wealth of knowledge and experience accumulated [1]. This is the stage in life when the concept of life satisfaction comes into the picture. It is also the time when various factors such as family dynamics and marriage start affecting the mental health of an individual. Let us discuss these in relation to our study of married individuals in Istanbul.

Demographic aspects of Istanbul

In Istanbul, in the past five years, the middle-aged and elderly have made up 21.9% of the population, and they are aging at the fastest rate in the world. The patriarchal family structure and regard for elders also continue to play significant roles in Istanbul society. The situation of the older and middle-aged population, who have seen the effects of industrialization and modernization, is also problematic. Istanbul's population has been showing an increasing trend since 2020, reaching approximately 15 million in 2022, which has increased by 1.43% from 2021. Most of the senior generation's resources and efforts were directed toward their offspring and the advancement of society [2].

The concept of life satisfaction

One of the key measures of quality of life is life satisfaction, which is defined as "a cognitive and judging process and a thorough assessment of the human quality of life." Life satisfaction in the middle and later years, along with mood and psychological well-being, can be seen as a major indicator of successfully adapting to the aging process. According to Medley, life satisfaction is an attitude and a subjective sensation of contentment and fulfillment over one's entire life. Additionally, he asserted that to feel content and happy with life before old age and to lead a life that is socially desirable in old age, the idea of successful aging is one that is meticulously fashioned by each person's ideas and self-concepts over the course of a significant period of time [3].

Family dynamics and marriage

Family and family values have an overall impact on people's lives both directly and indirectly. The psychological well-being of a couple increases with how well they get along [4] and life satisfaction rises with the quality of a parent-child connection [5].

Academics generally concur that challenging personal experiences are a significant contributor to the global growth of depression in women [6]. Marriage is one of the most important socially destructive factors in any society on Earth, and research has shown that it is associated with problems with women's mental health, like depression [7]. Previous studies looked at whether negative marital events, including emotional and sexual infidelity and the possibility of divorce, led to psychological problems like depression when taking into consideration the likelihood of marital conflict. The issue is still controversial and open to debate, despite the numerous studies that have been done in the past to specifically explore this area. Although the information has been gathered from numerous nations and people, many questions remain unanswered. Marriage and divorce may be considered culturally dangerous [6]. In the current situation, it is crucial to comprehend how this complex issue will affect Istanbul's culture and to create new approaches to dealing with complex phenomena.

The findings from the aforementioned studies imply that a thorough investigation of the problem of marital satisfaction in Istanbul is necessary. Examining and comprehending the implications associated with "infidelity" calls for a significant undertaking that accounts for the cultural complexity of Istanbul. Only a few earlier studies were able to relate the problem to women's violence and marital satisfaction in Istanbul [8].

Contributing factors of depression

High levels of attachment depression set apart preoccupied and anxious attachment types. High attachment depression sufferers frequently doubt their partner's commitment to the relationship and exhibit sensitive emotions when they are rejected [9]. In addition to having sensitive emotions, people with high attachment depression struggle to process their negative feelings and use coping mechanisms when faced with psychologically stressful situations [10]. Individuals with high depression then experience relational discomfort that is increased due to their impaired ability to control the negative affect [11].

It was also found that depression and certain life events, such as marriage style, education, and having children, were associated. Not only can marriage style, education, and having children affect the association between depression and life satisfaction, but they also have an impact on it [12].

Similar earlier studies showed that women who had experienced their husband's infidelity or threats to leave the marriage were six times more likely to be diagnosed with significant depression and anxiety than those who had not [13]. It was interesting to learn that individuals who divorced their relationships due to infidelity suffered from low self-esteem, fury, low confidence, loss of trust, and social isolation [8]. The results of multiple earlier studies demonstrate a significant correlation between stress and infidelity. Previous studies' findings suggested that unfaithful couples were more disturbed and worried than faithful ones. Finally, the researchers noted while focusing on depression and solitude that the main source of stress that results in psychological suffering is loneliness [14]. Similar research showed that 75% of persons who felt lonely believed that depressive symptoms were to blame for their loneliness [15]. Previous research has shown that, compared to other divorces, infidelity-related separations usually leave divorced individuals more predisposed to stress. Additionally, this study clarified that marriage style, having children, and education cause psychological issues. Similar research conducted in the past discovered that women are more likely than their male spouses to experience stress and depression. The present study sets out to determine the underlying effects of two theoretically sound but previously unpackaged elements on the psychological adjustment of married couples and divorcees.

Our goal is to understand the factors that prevent someone from becoming mentally healthy. So, the idea is to understand what factors in their life lead to depression (identify the causes of depression) so we can work on preventing it for future generations. In this paper, we seek to understand the effect of marriage style, having children, and attaining education on the prevalence of depression among married individuals living in Istanbul.

## Materials and methods

The article's research methodology is described in this section. We go into every step we take to create our models in complete detail, including the data we use, sample training and testing, and sample evaluation. The statistical analysis was conducted using the Anaconda 3-2019 package (Python programming language) and IBM SPSS version 21 (IBM Corp., Armonk, NY).

Objectives

Do factors like gender, education, working status, marriage style, and status of having a child relate to the prevalence of depression in married individuals in Istanbul?

Hypothesis

H1: There is a significant relationship between the occurrence of depression and factors such as gender, education, working status, marriage style, and status of having a child in married couples.

H2: There is no significant relationship between the occurrence of depression and factors such as gender, education, working status, marriage style, and status of having a child in married couples.

Research design

The study was a cross-sectional study using quantitative methods. It included independent variables, including the marriage style, the status of having a child, and education, and the dependent variable depression. For the purpose of this study, we defined "education" as "the act of teaching or learning general knowledge, honing one's thinking and judgment skills, and overall, intellectually preparing oneself or others for a mature life."

Also, we operationally thought of "depression" as "major depressive disorder, or depression that is a prevalent and dangerous medical condition, which has an adverse effect on one's emotions, thoughts, and behavior. Sadness and/or a loss of interest in previously pleasurable activities are major symptoms of depression."

Sample

The study included 433 subjects from Istanbul, and the details are mentioned further in the article.

As the population is finite but large in number, convenience sampling was adopted for the study. There are several approaches to determine the sample size. These include a census of small populations, imitating a sample size of similar studies, using published tables, and applying formulae to calculate a sample size. For populations that are large, Cochran developed the equation given below:

n_0_=$\frac{z^2pq}{e^2}$.

Here, *n_0_* is the sample size, *z^2^* is the abscissa of the normal curve that cuts off an area at the tails (1-equals the desired confidence level, e.g., 95%), *e* is the desired level of precision, *p* is the estimated proportion of an attribute that is present in the population, and *q* is *1-p*. The value of *z* is found in statistical tables, which contain the area under the normal curve. In this study, we presume that population size is finite and unknown; the formula was applied to know the sample size and the sample size was 433.

Search strategy and article selection

Our study was based on articles from ScienceDirect. Search terms included mental health, married couples, and depression. We modified the phrase prognosis to prediction after analyzing the initial batch of publications to find more papers that were pertinent. Based on the title and abstract, we only read the language that was pertinent to the study. These data were taken because Istanbul has a high rate of depression when examining married people. The data were taken only among married individuals in this study. We examined the connection between married individuals’ moods and potential prognoses. Age-specific information was not included in this study; instead, a subgroup of participants was examined [16].

Study area and participants

The samples were taken from Istanbul. The dataset included 433 participants' records. Our focus was on examining the existence of depression on married individuals in Istanbul and their risk factors.

Data acquisition

The Kaggle database was used to collect the data [17] and this investigation was carried out. The procedure explains how data are produced. The database is being set up for modeling in this step. Data about depression among married couples in Istanbul are taken as original datasets from Kaggle's websites. The database records served as the foundation for the implementation of every component. All demographic traits are related to these traits. Observations with missing values were then entirely subtracted from the features that had been chosen. Information from both structured and unstructured text statements was combined with the data. The forecast model was developed using a classification algorithm that forecasts hidden data. Since information about medical appointments was not included, our focus was on how depression developed in married couples.

Descriptive analysis

**Figure 1:**
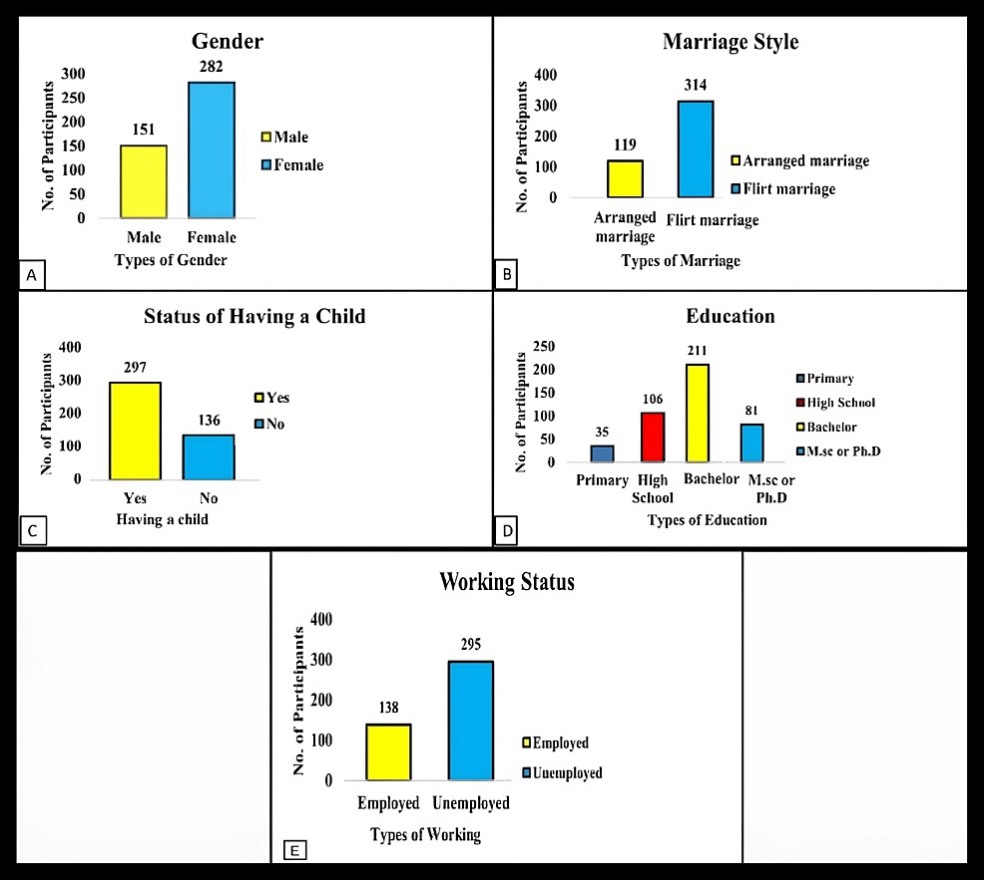
Descriptives of data The above figure shows the following distribution of data: A: Gender: male, n = 151; female, n = 282. B: Marriage style: arranged marriage, n = 119; love marriage, n = 314. C: Status of having a child: yes, n = 297; no, n = 136. D: Education: primary, n = 35; high school, n = 106; bachelor/graduation, n = 211; M.Sc. or Ph.D./postgraduation & above, n = 81. E: Working status: employed, n = 138; unemployed, n = 295. Note: All scales in the current study had satisfactory Cronbach's alpha coefficients (α = 0.877).

The dataset included records of 433 participants who were all from Istanbul. The details from the descriptive analysis are shown in Figure 1.

Beck Depression Inventory

The Beck Depression Inventory (BDI) is a 21-item, self-report rating inventory that measures characteristic attitudes and symptoms of depression [18]. The BDI has been developed in different forms. The BDI takes approximately 10 minutes to complete, although clients require a fifth to sixth-grade reading level to adequately understand the questions. The 21 categories are given in the Appendix. They are referred to as B1 to B21 in this paper [18].

The mean, standard deviation, maximum, and minimum of depression in married couples' BDI scores are shown in Table 1.

**Table 1:** Beck Depression Inventory score

	Beck Depression Inventory score
Count	433
Mean	10.337182
Std.	9.117135
Min.	<0.00001
25%	3.000000
50%	8.000000
75%	15.000000
Max.	61.000000

Correlation heatmap

**Figure 2:**
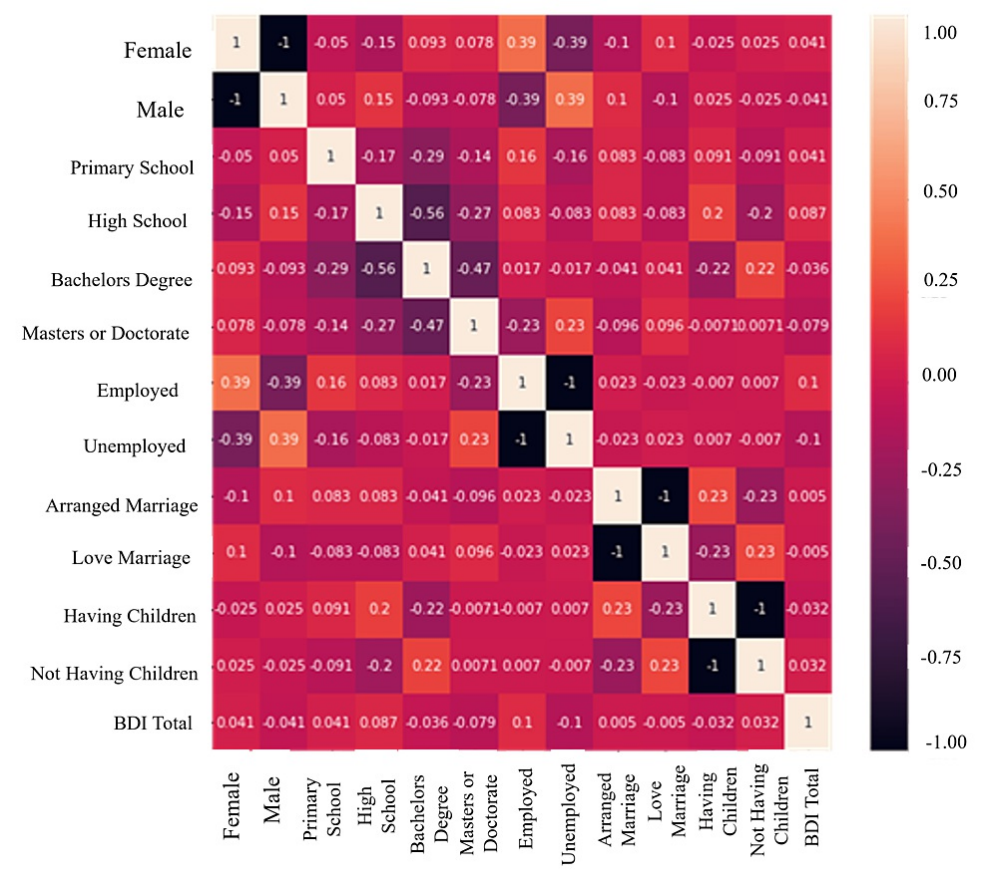
Performance of the correlation heatmap BDI: Beck Depression Inventory.

Heatmap is utilized to display the correlation between features of a dataset. The performance of the correlation heatmap is shown in Figure 2.

The correlation between characteristics is calculated using Pearson correlation, and the formula is given below:

$$R_{i,j}=Cov(i,j)/ \sqrt (Var(i) Var(j)$$

Where *i* and *j* denote vector, *Cov(i,j)* denotes covariance of *i* and *j*, and *Var(i)* and *Var(j)* denote standard deviation of *i* and *j*.

The correlation coefficient has a value range of -1 to 1. The stronger the correlation between the two vectors, the higher the correlation coefficient.

The data are structured in the form of a heatmap, in which the percentage of truly classified events is shown in the light color and the percentage of incorrectly classified events in the dark color (Figure 2). Understanding the effects of the features included in the best model for depression in married couples by explaining the effectiveness of the best model. There is a strong correlation between education, gender, the status of having a child, marriage style, and working status. That is, the correlation between working status and gender is 0.39, the correlation between education and the status of having a child is 0.22, and the correlation between marriage style and the status of having a child is 0.23.

Performance comparison between the model summary of the regression analysis

For regression analysis, the dependent variable is depression and the independent variables are the marriage style, status of having a child, types of working, and education. The R, R square, adjusted R square, and standard error of the estimate were calculated to compare the groups of depression in married couples that were gender, education, working status, marriage style, and status of having a child for study participants of the Istanbul population and are shown in Table 2 given below.

**Table 2:** Model summary of the regression analysis ^a^ Dependent variable.

Variables	R	R square	Adjusted R square	Std. error of the estimate
Gender	.316^a^	.100	.054	.464
Education	.246^a^	.061	.013	.837
Working status	.300^a^	.090	.043	.456
Marriage style	.206^a^	.042	-.007	.448
Status of having a child	.222^a^	.049	.001	.465

Comparing the values of the five demographic variable groups to each other, the gender group has the highest value of R (0.316), R square (0.100), and adjusted R square (0.054), and its standard error of estimated value (0.448) is the lowest value of marriage style.

Performance comparison between ANOVA of the regression analysis

In Table 3, the ANOVA values are compared between the three groups in five demographic categories where the significant value is less than 0.05 (p < 0.05). So here all five groups selected have significant values.

**Table 3:** ANOVA table for significant value of the regression analysis ^a^ Dependent variable. * Significant value (p < 0.05). ** Significant value (p > 0.05).

ANOVA						
Variables		Sum of squares	Df	Mean square	F	p
Gender	Regression	9.817	21	.467	2.170	.002^*^
	Residual	88.525	411	.215	-	-
	Total	98.342	432	-	-	-
Education	Regression	18.527	21	.882	1.261	.197^**^
	Residual	287.630	411	.700	-	-
	Total	306.157	432	-	-	-
Working status	Regression	8.453	21	.403	1.933	.008^*^
	Residual	85.566	411	.208	-	-
	Total	94.018	432	-	-	-
Marriage style	Regression	3.659	21	.174	.867	.635^**^
	Residual	82.636	411	.201	-	-
	Total	86.296	432	-	-	-
Status of having a child	Regression	4.588	21	.218	1.012	.447^**^
	Residual	88.696	411	.216	-	-
	Total	93.284	432	-	-	-

Performance comparison between significant coefficients of the regression analysis

Based on the regression model, we determined the coefficient value and its associated significance level for each predictor. The variables B1 to B21 refer to the 21 categories in the BDI (Appendix). The coefficient of significance value for each gender group is compared in Table 4, where significant values are those with p-values of less than 0.05 (p < 0.05) for all variables.

**Table 4:** Comparing the coefficient’s significant value of the gender group ^a^ Dependent variable: gender. * Significant value (p < 0.05). ** Significant value (p > 0.05).

Coefficients					
Variables	Unstandardized coefficients		Standardized coefficients	T	P
	B	Std. error	Beta		
Constant	1.417	.040		35.825	.000*
B1	-.025	.042	-.039	-.584	.560**
B2	.031	.039	.048	.792	.429**
B3	.098	.036	.171	2.724	.007*
B4	.013	.044	.018	.284	.777**
B5	-.005	.049	-.007	-.104	.917**
B6	.061	.031	.108	1.953	.051*
B7	-.049	.047	-.066	-1.053	.293**
B8	-.069	.045	-.098	-1.516	.130**
B9	.040	.060	.040	.674	.501**
B10	-.006	.026	-.013	-.228	.820**
B11	-.018	.032	-.034	-.571	.568**
B12	-.058	.038	-.095	-1.519	.129**
B13	.069	.038	.110	1.794	.074**
B14	-.010	.045	-.014	-.225	.822**
B15	-.031	.040	-.052	-.787	.432**
B16	-.048	.036	-.074	-1.342	.180**
B17	-.070	.041	-.106	-1.708	.088**
B18	.078	.048	.094	1.627	.105**
B19	.041	.042	.054	.996	.320**
B20	.034	.039	.050	.877	.381**
B21	-.065	.035	-.100	-1.822	.069**

According to these findings, there was no statistically significant difference between the gender groups for a significance value greater than 0.05 (p > 0.05).

In Table 5, the education group's coefficient of significance is compared, with some significant values (those with p-values less than 0.05 (p < 0.05)) in all variables. According to the findings, the education group's difference was not statistically significant when the significant value was more than 0.05 (p > 0.05).

**Table 5:** Comparing the coefficient’s significant value of the education group ^a^ Dependent variable: education. * Significant value (p < 0.05). ** Significant value (p > 0.05).

Coefficients										
Variables		Unstandardized coefficients				Standardized coefficients	T		P	
		B		Std. error		Beta				
Constant	2.804		.071				39.324	.000*		
B1	.011		.077		.010		.143	.887**		
B2	-.061		.071		-.054		-.859	.391**		
B3	-.078		.065		-.077		-1.204	.229**		
B4	-.021		.080		-.016		-.259	.796**		
B5	.099		.089		.073		1.106	.269**		
B6	-.072		.056		-.073		-1.282	.200**		
B7	.080		.084		.061		.950	.343**		
B8	.045		.081		.037		.557	.578**		
B9	-.036		.108		-.020		-.335	.737**		
B10	-.077		.047		-.093		-1.637	.102**		
B11	-.058		.058		-.062		-.998	.319**		
B12	.095		.069		.088		1.373	.171**		
B13	-.059		.069		-.053		-.849	.396**		
B14	.101		.080		.078		1.252	.211**		
B15	-.045		.071		-.043		-.638	.524**		
B16	-.013		.065		-.011		-.202	.840**		
B17	.112		.074		.096		1.506	.133**		
B18	-.108		.086		-.074		-1.259	.209**		
B19	.037		.075		.028		.498	.619**		
B20	-.013		.070		-.011		-.191	.848**		
B21	-.121		.064		-.106		-1.888	.060**		

The working status group's coefficient of significance is compared in Table 6, with some significant values (those with p-values less than 0.05 (p < 0.05)) for all variables. Thus, a significant value smaller than 0.05 is chosen in this instance. According to the findings, there was no significant difference between the working status group, as the significant value is larger than 0.05 (p > 0.05).

**Table 6:** Comparing the coefficient’s significant value of the working status group ^a^ Dependent variable: working status. * Significant value (p < 0.05). ** Significant value (p > 0.05).

Coefficients										
Variables		Unstandardized coefficients				Standardized coefficients	T		P	
		B		Std. error		Beta				
Constant	1.738		.039				44.695	.000*		
B1	-.047		.042		-.075		-1.116	.265**		
B2	-.011		.039		-.018		-.294	.769**		
B3	.031		.035		.055		.866	.387**		
B4	-.017		.043		-.025		-.397	.691**		
B5	.035		.049		.047		.720	.472**		
B6	.055		.031		.100		1.796	.073**		
B7	-.065		.046		-.090		-1.423	.155**		
B8	.007		.044		.011		.167	.868**		
B9	.034		.059		.035		.584	.559**		
B10	-.010		.026		-.022		-.392	.695**		
B11	-.028		.031		-.055		-.905	.366**		
B12	-.096		.038		-.159		-2.532	.012*		
B13	.073		.038		.120		1.944	.053*		
B14	.051		.044		.072		1.170	.243**		
B15	-.099		.039		-.167		-2.537	.012*		
B16	-.067		.035		-.105		-1.895	.059*		
B17	.019		.040		.030		.476	.634**		
B18	.015		.047		.018		.311	.756**		
B19	.052		.041		.070		1.277	.202**		
B20	.039		.038		.058		1.013	.312**		
B21	-.005		.035		-.007		-.133	.894**		

In Table 7, the marriage style group's coefficient of significant value is compared, with some significant values being less than 0.05 (p < 0.05) in all variables. Thus, a significant value smaller than 0.05 is chosen in this instance. According to the findings, there was no discernible difference between the marriage type group and the significant value was more than 0.05 (p > 0.05).

**Table 7:** Comparing the coefficient’s significant value of the marriage style group ^a^ Dependent variable: marriage style. * Significant value (p < 0.05). ** Significant value (p > 0.05).

Coefficients^a^					
Variables	Unstandardized coefficients		Standardized coefficients	T	p
	B	Std. error	Beta		
Constant	1.710	.038		44.735	.000*
B1	-.034	.041	-.057	-.836	.403**
B2	-.017	.038	-.028	-.441	.660**
B3	.031	.035	.058	.891	.374**
B4	.003	.043	.005	.081	.935**
B5	-.011	.048	-.015	-.224	.823**
B6	.021	.030	.039	.689	.491**
B7	-.005	.045	-.007	-.111	.911**
B8	-.002	.044	-.003	-.048	.961**
B9	-.019	.058	-.021	-.337	.736**
B10	-.039	.025	-.089	-1.556	.121**
B11	.054	.031	.108	1.743	.082**
B12	.087	.037	.151	2.337	.020*
B13	-.029	.037	-.049	-.785	.433**
B14	.011	.043	.017	.263	.793**
B15	-.063	.038	-.112	-1.656	.099**
B16	-.014	.035	-.023	-.409	.682**
B17	-.005	.040	-.007	-.117	.907**
B18	-.011	.046	-.014	-.230	.818**
B19	.058	.040	.081	1.442	.150**
B20	-.025	.038	-.038	-.650	.516**
B21	.016	.034	.027	.473	.636**

Table 8 compares the coefficient of significance among the groups of people who report having children, where some significant values are less than 0.05 (p < 0.05) for all variables. Thus, a significant value smaller than 0.05 is chosen in this instance. According to the findings, there was no statistically significant difference between the status of having a children group when the significant value was larger than 0.05 (p > 0.05).

**Table 8:** Comparing the coefficient’s significant value of the status of having a child group ^a^ Dependent variable: status of having a child. * Significant value (p < 0.05). ** Significant value (p > 0.05).

Coefficients^a^					
Variables	Unstandardized coefficients		Standardized coefficients	T	P
	B	Std. error	Beta		
Constant	1.333	.040		33.671	.000*
B1	.041	.043	.065	.954	.340**
B2	.050	.039	.079	1.263	.207**
B3	.034	.036	.061	.944	.346**
B4	.026	.044	.038	.595	.552**
B5	-.038	.050	-.051	-.772	.440**
B6	-.007	.031	-.012	-.218	.827**
B7	-.015	.047	-.021	-.331	.741**
B8	.075	.045	.110	1.651	.099**
B9	-.026	.060	-.027	-.438	.661**
B10	-.012	.026	-.026	-.457	.648**
B11	-.035	.032	-.068	-1.092	.275**
B12	.041	.038	.069	1.077	.282**
B13	-.016	.038	-.026	-.421	.674**
B14	-.051	.045	-.072	-1.140	.255**
B15	-.011	.040	-.019	-.287	.774**
B16	-.029	.036	-.046	-.818	.414**
B17	-.046	.041	-.072	-1.127	.260**
B18	.072	.048	.090	1.508	.132**
B19	-.002	.042	-.002	-.042	.966**
B20	.041	.039	.062	1.058	.291**
B21	-.061	.035	-.098	-1.721	.086**

Machine learning application to predict depression in married couples

The machine learning (ML) model is a representation illustrating the development process [19].

We used two ML approaches for categorization - the decision tree (DT) and random forest (RF) - to pinpoint the crucial factors linked to depression in married couples. This model was chosen to be implemented in this study because it gives the highest accuracy value.

Decision Tree

A DT is a flowchart that resembles a tree and creates a binary tree. The DT method works best for the classification issue. A DT is a supervised learning technique, which means that the data used to build the tree already knows the solutions. The level of classification accuracy reached on the training dataset and the size of the tree have the biggest effects on the model's ability to predict marital sadness. An innovative technique for building classification models from a set of training data is the DT algorithm. To create DT architectures, top-down layered dividing and conquering strategies are employed. The framework includes training data modeling for nodes and branches. The root node, which is the first node, divides up each bit of data until a termination condition is satisfied.

The root node (parent node), internal node (child node), and leaf node (terminating node) are the three structural components that make up the DT. The leaf node (terminating node), the end node, completes the DT whereas the internal node (child node) indicates the qualities that are contained within the tree. The DT terminating criteria say that all samples for a particular node belong to the same type of class and that there are no residual attributes for further splitting. There are many distinct types of DT, but the most well-known ones are information gain (IG), Gini index (GI), and gain ratio (GR). A DT can be produced using the algorithms ID3, J-48, C4.5, and C5.0. The most well-known algorithms are those in C5.0. The decision rule was cut down, and the DT was made more compact, using the pruning technique [20].

Random Forest

As part of the ensemble learning model, the classification technique known as RF combines predictions from inferior classifiers. It builds an indicator ensemble out of DTs that are growing according to discrete ensemble parameters in a data subspace that is randomly chosen [21]. It produces exceptionally accurate predictions, can handle a huge number of input variables without over-fitting, and is rapid and easy to use. Making a variety of trees that will each help in casting a vote for a specific class is the first step of the procedure. To cast a vote, the training data must be divided into smaller, equal subsets before a DT is created. The tree is built using the RF algorithm.

In the data collection, let X represent the number of classes and Y represent the number of variables. The tree node is evaluated using the input variable *y*. With a value for the *y* variable that is picked at random, determine the best split for each tree node. The tree is finally fully grown and no longer requires pruning. A new sample is projected when the tree is uprooted. At the terminal node, the label is given to the training sample. The RF forecast is then observed after several iterations of this procedure across all trees [22].

Performance Assessment

$$(1) Accuracy= \frac{True_{positive} + True_{negative}}{True_{positive} + True_{negative} +False_{positive} +False_{negative}}$$

$$(2) Precision=\frac{True_{positive}}{True_{positive} + False_{positive}}$$

$$(3) Recall= \frac{True_{positive}}{True_{positive} + False_{negative}}$$

$$(4) F1score=\frac{2}{\frac{1}{Precision}+ \frac{1}{Recall}} = \frac{2*(Average Precision*Average Recall)}{(Average Precision+ Average Recall)}$$

Where the percentage of times the classifier successfully identified a positive sample as positive is expressed as true positives (TP). The percentage of times the classifier correctly identified a negative sample as negative is known as true negatives (TN). False positive (FP) describes the frequency with which a negative sample was mistakenly classified as a positive sample by the classifier. False negative (FN) refers to the frequency with which a positive sample was incorrectly categorized as a negative by the model. The efficiency and efficacy of the faster DT and RF model were assessed for both the testing and validation sets using the accuracy (A), recall (R), precision (P), and F1 score (F1).

Model Assessment (Validation and Comparison of the Models)

To more accurately evaluate model performance and minimize any potential deviation between the estimations, we employed five-repeated five-fold cross-validation. The data are split into two subsets using this technique - training and test data. The model is developed using the training data and assessed using the test dataset. The entire performance indices, such as precision, recall, F1 score, support, and accuracy, are created using the performance estimations from five iterative cross-validations. It is important to keep in mind that the ratio of cases to controls in each subset remained unchanged. Each subgroup gave an accurate picture of the state of the larger community as well as the main sample.

Two sets of testing and training sets of data were created. The data were split into two distinct situations, the train and test sets being, respectively, 70% and 30% for situation 1 and 80% and 20% for situation 2. We have shared the results for situation 1 below.

Table 9 shows the results of the prediction of depression in married couples using the RF model. We can observe that the classifiers predicted depression with an accuracy of around 60% and an F1 score of around 75%. Precision and recall range between 60% and 83%. Furthermore, the table reveals that the performance of the classifiers is almost similar in terms of predicting depression.

**Table 9:** Results of the prediction of depression in married couples using the random forest model

Model	Accuracy (%)	F1 score (%)	Precision (%)	Recall (%)
Random forest	60	75	60	82.5
Macro average	-	12	10	17
Weighted average	-	45	36	60

Table 10 shows the results of the prediction of depression in married couples using the DT model. We can observe that the classifiers predicted depression with an accuracy of around 80% and an F1 score of around 85%. Precision and recall range between 72% and 96%. Furthermore, the table reveals that the performance of the classifiers is almost similar in terms of predicting depression.

**Table 10:** Results of the prediction of depression in married couples using the decision tree model

Model	Accuracy (%)	F1 score (%)	Precision (%)	Recall (%)
Decision tree	80	85	72	96
Macro average	-	13	12	15
Weighted average	-	44	38	53

## Results

Table 9 demonstrates the results when the RF technique is used for predicting depression among married couples. F1 score accounts for 75% of the total number of different approaches, precision accounts for 60%, accuracy of RF accounts for 60%, and recall accounts for 82.5%. Table 10 demonstrates the results when the DT technique is used for predicting depression among married couples. F1 score accounts for 85% of the total number of different approaches, precision accounts for 72%, accuracy of DT accounts for 80%, and recall accounts for 96%. Finally, Tables 9, 10 demonstrate that DT is 20% more accurate than RF models.

Regarding *H1*, which is supported by the current study, it claims that "there is a significant relationship between the occurrence of depression and factors such as gender, education, working status, marriage style, and status of having a child in married couples." According to the findings presented in Tables 9, 10, depression among married couples was positively correlated with factors such as gender, education, working status, marriage style, and status of having a child. Regarding *H2*, which is not supported by the current study, it claims that "there is no significant relationship between the occurrence of depression and gender, education, working status, marriage style, and status of having a child in married couples." According to the findings presented in Tables 4-8, depression among married couples was positively correlated with factors such as gender, education, working status, marriage style, and status of having a child.

## Discussion

The goal of the current study is to research the moderating effects of gender, education, employment position, marital status, and status of having children on the development of depressive married couples. The findings presented in Tables 4-8 showed that factors such as gender, education, working status, marriage style, and status of having a child were moderators of melancholy among married couples.

The results of the present study support some of those of earlier ones [23,24]. Also, some previous research showed that divorced people had higher levels of anxiety, stress, and depression than married people. Additionally, previous studies have demonstrated that married women with solid relationships with their husbands and families were less likely to have psychological issues like stress, anger, anxiety, loneliness, and depression [25]. However, other studies found that after having a divorce from their partners, women were more likely to commit emotional and sexual adultery [26,27].

Finally, it was also indicated by the researchers that loneliness is the major cause of one’s stress, which causes psychological distress. While examining isolation and depression in particular [28], similar studies found that 75% of persons feeling isolation believed that it was a result of depressive symptoms [29].

Depression in the elderly is often accompanied by other health problems or life events, and most of the depressive symptoms are considered a natural reaction due to aging and are often neglected or overlooked. Depression in middle age and old age tends to consist of more depressive symptoms as the number of health problems increases, and life events that cause depression include job loss, retirement, death of a spouse, divorce, and loss of economic power [30].

Strengths

Firstly, the study demonstrates the use of ML techniques to analyze the data and predict depression in married individuals. This is not used commonly in the medical literature. This paves the way for future research that can apply ML techniques to predict the occurrence of a medical condition and proactively provide the best possible medical care to the patients. Secondly, our study also has a reasonably large sample size, which adds to the reliability of the study.

The results of the study are a valuable addition to medical literature.

Limitations

Our study has the following limitations. First, we were only able to conduct the study in Istanbul, where the available sample group was too small to represent all married individuals in Istanbul. Second, this study could not identify the impact of other diseases.

Implications of the study

Researchers and clinicians have not yet developed any strict guidelines for preventing depression, and more research is needed to eradicate this societal ill. This study could aid psychologists in predicting the occurrence of depression and reducing the effects of depression. The study's findings should emphasize the females. The results of the study may help clinicians work on the root causes of unhappiness before it results in divorce and depression. The study attempted to emphasize the factor of depression in married couples. By concentrating on the factors that contribute to depression and the solutions available, future studies may be further aided.

## Conclusions

The finding of this current study illustrates that compared to married couples, individuals with marriage styles and who have a child have more psychological problems. The results of this study elaborate that compared to arranged married couples, people who do love marriage are more prone to psychological problems. Also, the result clearly shows that depression is more common in females than males. The findings indicate that when sadness is either mental or physical, unemployed people are more depressed than married couples. The findings also indicate that single students experience depression at a higher rate than married people. It is believed that this study would aid future researchers in examining some of the subject's unexplored facets. It is hoped that this study will enable future researchers to explore some more unidentified aspects of the subject.

## Appendices

Beck’s Depression Inventory

Sociodemographic details are to be filled by all participants and include the details mentioned in Table 11 as shown below.

**Table 11:** Sociodemographic details

Name	
Marital status	
Age	
Sex	
Occupation	
Education	

Instructions

The questionnaire consists of 21 groups of statements. Please read each group of statements carefully, and then pick out the one statement in each group that best describes the way you have been feeling during the past two weeks, including today. Circle the number beside the statement you have picked. If several statements in the group seem to apply equally well, circle the highest number for that group. Be sure that you do not choose more than one statement for any group, including item 16 (Changes in Sleeping Pattern) or item 18 (Changes in Appetite).

1. Sadness

0: I do not feel sad.

1: I feel sad much of the time.

2: I am sad all the time.

3: I am so sad or unhappy that I can't stand it.

2. Pessimism

0: I am not discouraged about my future.

1: I feel more discouraged about my future than I used to be.

2: I do not expect things to work out for me.

3: I feel my future is hopeless and will only get worse.

3. Past failure

0: I do not feel like a failure.

1: I have failed more than I should have.

2: As I look back, I see a lot of failures.

3: I feel I am a total failure as a person.

4. Loss of pleasure

0: I get as much pleasure as I ever did from the things I enjoy.

1: I don't enjoy things as much as I used to.

2: I get very little pleasure from the things I used to enjoy.

3: I can't get any pleasure from the things I used to enjoy.

5. Guilty feelings

0: I don't feel particularly guilty.

1: I feel guilty over many things I have done or should have done.

2: I feel quite guilty most of the time.

3: I feel guilty all of the time.

6. Punishment feelings

0: I don't feel I am being punished.

1: I feel I may be punished.

2: I expect to be punished.

3: I feel I am being punished.

7. Self-dislike

0: I feel the same about myself as ever.

1: I have lost confidence in myself.

2: I am disappointed in myself.

3: I dislike myself.

8. Self-criticalness

0: I don't criticize or blame myself more than usual.

1: I am more critical of myself than I used to be.

2: I criticize myself for all of my faults.

3: I blame myself for everything bad that happens.

9. Suicidal thoughts or wishes

0: I don't have any thoughts of killing myself.

1: I have thoughts of killing myself, but I would not carry them out.

2: I would like to kill myself.

3: I would kill myself if I had the chance.

10. Crying

0: I don't cry any more than I used to.

1: I cry more than I used to.

2: I cry over every little thing.

3: I feel like crying, but I can't.

11. Agitation

0: I am no more restless or wound up than usual.

1: I feel more restless or wound up than usual.

2: I am so restless or agitated that it's hard to stay still.

3: I am so restless or agitated that I have to keep moving or doing something.

12. Loss of interest

0: I have not lost interest in other people or activities.

1: I am less interested in other people or things than before.

2: I have lost most of my interest in other people or things.

3: It's hard to get interested in anything.

13. Indecisiveness

0: I make decisions about as well as ever.

1: I find it more difficult to make decisions than usual.

2: I have much greater difficulty in making decisions than I used to.

3: I have trouble making any decisions.

14. Worthlessness

0: I do not feel I am worthless.

1: I don't consider myself as worthwhile and useful as I used to.

2: I feel more worthless as compared to other people.

3: I feel utterly worthless.

15. Loss of energy

0: I have as much energy as ever.

1: I have less energy than I used to have.

2: I don't have enough energy to do very much.

3: I don't have enough energy to do anything.

16. Changes in sleeping pattern

0: I have not experienced any change in my sleeping pattern.

1 - a. I sleep somewhat more than usual.

b. I sleep somewhat less than usual.

2 - a. I sleep a lot more than usual.

b. I sleep a lot less than usual.

3 - a. I sleep most of the day.

b. I wake up one to two hours early and can't get back to sleep.

17. Irritability

0: I am no more irritable than usual.

1: I am more irritable than usual.

2: I am much more irritable than usual.

3: I am irritable all the time.

18. Changes in appetite

0: I have not experienced any change in my appetite.

1 - a. My appetite is somewhat less than usual.

b. My appetite is somewhat greater than usual.

2 - a. My appetite is much less than before.

b. My appetite is much greater than usual.

3 - a. I have no appetite at all.

b. I crave food all the time.

19. Concentration difficulty

0: I can concentrate as well as ever.

1: I can't concentrate as well as usual.

2: It's hard to keep my mind on anything for very long.

3: I find I can't concentrate on anything.

20. Tiredness or fatigue

0: I am no more tired or fatigued than usual.

1: I get more tired or fatigued more easily than usual.

2: I am too tired or fatigued to do a lot of the things I used to do.

3: I am too tired or fatigued to do most of the things I used to do.

21. Loss of interest in sex

0: I have not noticed any recent change in my interest in sex.

1: I am less interested in sex than I used to be.

2: I am much less interested in sex now.

3: I have lost interest in sex completely.

Scoring the Beck Depression Inventory

After you have completed the questionnaire, add up the score for each of the 21 questions. The following table (Table 12) indicates the relationship between total score and level of depression according to the Beck Depression Inventory.

**Table 12:** Scoring the Beck Depression Inventory

Classification	Total score	Level of depression
Low	1-10	Normal ups and downs
	11-16	Mild mood disturbance
Moderate	17-20	Borderline clinical depression
	21-30	Moderate depression
Significant	31-40	Severe depression
	Over 40	Extreme depression
